# Forward models of repetition suppression depend critically on assumptions of noise and granularity

**DOI:** 10.1038/s41467-020-18315-w

**Published:** 2020-09-18

**Authors:** Fernando M. Ramírez, Elisha P. Merriam

**Affiliations:** Laboratory of Brain and Cognition, National Institute of Mental Health, Bethesda, USA

**Keywords:** Computational biology and bioinformatics, Neural encoding

**Arising from** A. Alink et al. *Nature Communications* 10.1038/s41467-018-05957-0 (2018)

In a recent issue of *Nature Communications*, Alink et al.^1^ used computational models to adjudicate between competing neural mechanisms for repetition suppression. The authors compared the model’s output with functional magnetic resonance imaging (fMRI) measurements and concluded that repetition suppression^2–7^ is best modeled by local neural scaling. Here, we point out a coding error in defining the noise distribution. Correcting this error fundamentally changed their results. We show that models of the class implemented by Alink et al. are sensitive to a range of assumptions and parameters that affect the signal-to-noise ratio (SNR) of simulated brain patterns. We argue that unless such parameters are appropriately constrained, and the modeled SNR regime matched to empirical data, ensuing inferences regarding neural coding are inconclusive. Our observations have broad implications for the modeling of neural responses.

In analyzing responses from any neural system, it is common to parcellate components of the measurement into putative signal and noise, which are then parameterized by a formal model. Simulations of neural responses require explicit assumptions about the nature of signal and noise components. In generalized linear models of fMRI time series, the error term, *ε*, is used to denote zero-mean Gaussian noise^8^. Consistent with this, the magnitude of the noise added by Alink et al. to simulated brain activation patterns was controlled by a parameter, *σ*_Noise_, which was set to a value of 0.1. However, the implementation of their noise model is problematic. First, the magnitude of the added noise is arbitrary; *σ*_Noise_ was set to 0.1 with no justification. Second, a signal with unit variance (cf. Peer review file) and a noise variance of 0.1 would imply an SNR of roughly 100. Empirically measured SNR values are typically much smaller^9^. Third, while the authors intended to add noise from a Gaussian distribution with a mean of 0 and standard deviation of 0.1, they actually added noise from a uniform distribution with a mean of 0.05 and a standard deviation of 0.029. This error created a 12-fold reduction in the noise variance, leading to model SNRs as high as 963, which are currently unattainable with fMRI. Fourth, if the mean of the noise distribution is not zero, this will evidently affect the direction of simulated pattern vectors formed by the addition of a signal and a noise component^10^.

We reran Alink et al.’s simulations after correcting this error. The “winning” model reported for the gratings dataset no longer captured three of the six data-features it was intended to reproduce (Fig. 1). In fact, none of the 25 local-scaling models identified in their Supplementary Table 1 matched the totality of the empirically observed data features. Thus, while Alink et al. state that one model fits better than the rest, our simulations challenge this conclusion.

**Fig. 1:**
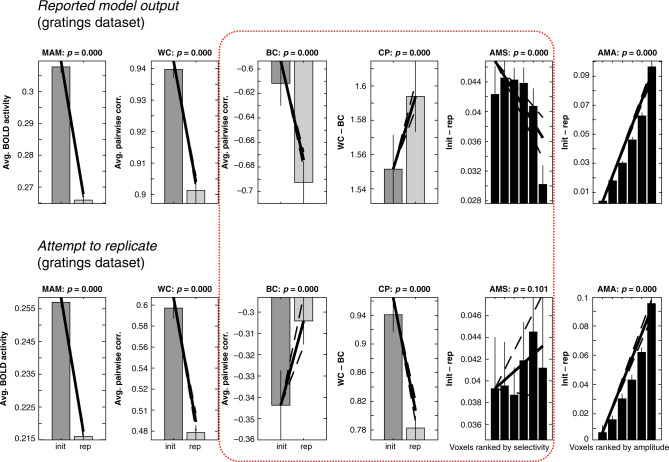
Noise parameters influence feed-forward models of fMRI-pattern correlations. Top row: pattern of results reported by Alink et al. for the winning parameter combination [*a* = 0.8, *b* = 0.4, *σ* = 0.4] for the grating dataset (cf. Supplementary Fig. 4 in Alink et al.). The direction (increase or decrease when comparing initial and repeated responses) of the six empirically observed data-features (cf. Figure 3 in Alink et al.) is only observed when uniformly distributed noise in the interval [0, 0.1] is added to the simulated brain patterns. Bottom row: the qualitative pattern of results observed for the winning parameter combination shown in the top row changed substantially when adding zero-mean Gaussian noise with a standard deviation of 0.1 instead of uniformly distributed noise. Of particular interest, the data-features BC, CP, and AMS are (shown within a red box) no longer qualitatively consistent with the empirical observations. Compare the corresponding slopes of lines in the top and bottom rows. Init initial presentation, rep repeated presentation, MAM mean amplitude modulation, WC within-class correlation, BC between-class correlation, CP “classification performance” (CP = WC − BC), AMS amplitude modulation by selectivity, AMA amplitude modulation by amplitude. Solid bars indicate mean of each condition and error bars 95% confidence intervals given the (simulated) between-participant variability. Diagonal lines indicate the slope of linear contrasts across conditions, and dashed lines indicate 95% confidence interval of the slope. See Alink et al. for methodological details and proposed interpretation of error bars and *p* values above each subpanel. In our view, given that arbitrary modeling choices determine the across-subject variability produced by the model, the reported error bars and accompanying *p* values have therefore little, if any, statistical meaning.

We wondered if, after correcting the noise distribution, a different combination of model parameters might match the empirical observations, and if so, whether such a combination would still favor local scaling. We found none of the 648 local-scaling models defined by the search grid matched the six empirically observed data-features. Moreover, when we explored higher SNR regimes, local-sharpening models showed repetition suppression effects that, like the local-scaling model, also matched the six empirically observed data features according to Alink et al.’s criteria. Because the local-sharpening model *can* also reproduce all data features for the grating experiment, and because Alink et al.’s model is intrinsically biased in favor of the local-scaling model (see Supplementary Material), the alternative interpretation that local sharpening provides a better account of the data may seem supported. We argue, however, that inferences regarding neural properties based on forward models are invalid unless constrained by estimates of the SNR of the data, or, alternatively, a demonstration that the results are robust to a range of noise levels likely representative of the data. We estimated the SNR of the actual fMRI data made available by Alink et al. We observed empirical SNR values that were markedly smaller than those of the models able to reproduce the six data-features (see Supplementary Material). Finally, if two reasonably constrained models did turn out to fit the data, which we argue is not the case here, concluding that one model provides a better account of the data than the other would require formal model selection^11,12^, an endeavor not undertaken by Alink et al.

Having shown that assumptions about the noise affect the output of Alink et al.’s model, we wondered if the models are also dependent on assumptions regarding the strength of the signal^13,14^. We explored the impact of two key parameters of Alink et al.’s model: tuning bandwidth (*σ*_Tuning_) and granularity (*G*) (viz. *N*, using Alink et al.’s terminology). Figure 2 shows that signal strength depends on both parameters. In particular, we note that *σ*_Tuning_ is a free parameter in Alink et al.’s model. This implies that voxels belonging to models with narrower tuning widths will exhibit systematically lower signal levels than voxels with broader tuning functions. If competing models of repetition suppression are to be distinguished, candidate models must be matched with regard to their empirically observed pre-adaptation SNR regime. However, Alink et al.’s model imposes different SNR regimes across models, effectively favoring some models over others. To avoid this bias, the implemented models would require a flat surface (Fig. 2b, left). This is evidently not the case, as demonstrated by the clearly non-flat surface shown to the right under “Simulation results.” This dependency of SNR regime on tuning width is a structural limitation of Alink et al.’s model that cannot be corrected by parameter adjustments.

**Fig. 2:**
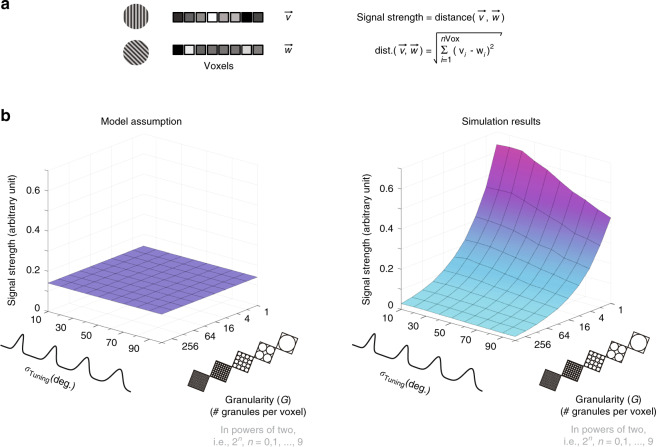
Noise amplitude and signal strength influence empirically observed fMRI–pattern correlations. **a** fMRI patterns formed by concatenating responses across voxels for each of two experimental conditions—here, visual gratings oriented either 45° or 90° from the horizontal. The strength of the signal component distinguishing the brain responses associated with these two gratings can be quantified as the Euclidean distance between these two spatially distributed brain response patterns, treated as vectors, and denoted here as $\vec v$ and $\vec w$. **b** Simulation results: signal strength as a function of tuning bandwidth and granularity. In the class of models implemented by Alink et al., the tuning bandwidth of feature-tuned neural populations has been parametrized by Gaussian distributions. The preferred orientation of each neural population is described by *μ*_Tuning_, while *σ*_Tuning_ describes how tightly tuned each population is about its preferred orientation. In turn, the level of granularity of simulated fMRI data has been controlled by a positive integer (*G*) specifying the number of similarly tuned neural clusters, here referred to as granules, assumed to be sampled by each voxel^13^. The 3D surface shown to the right under the label “Simulation results” clearly demonstrates that granularity (*x*-axis), as well as tuning width (*y*-axis), influence the strength of simulated fMRI patterns. For each admissible parameter combination of *G* and *σ*_Tuning_ the *z*-axis indicates the average strength (across 25 randomly seeded simulations) of the signal distinguishing the fMRI response patterns denoted by $\vec v$ and $\vec w$. The full range of simulated granularity levels is [1, 512] (2^*n*^, with *n* = 0, 1, …, 9 granules per voxel). A dramatic effect of granularity on signal strength can be noted along the *x*-axis. If granularity were irrelevant, the observed monotonically decreasing curve would be instead a flat line. Given that pairwise correlations are known to be determined by noise amplitude as well as signal strength, this simulation demonstrates that the validity of inferences regarding neural coding based on fMRI–pattern correlations depend on granularity assumptions as well as noise parameters.

A final concern regards the impact of granularity assumptions on the outcome of the class of models discussed here. Alink et al. asserted that changing the number of orientation-tuned sub-populations (or clusters, or granules) assumed to be sampled within each fMRI voxel “does not have a qualitative effect on the simulation results.” This statement is inconsistent with previous work^13^ that manipulated this parameter to change the granularity of simulated brain patterns. This work revealed that pattern correlations are indeed sensitive to such changes—as well as changes in other properties that influence SNR, such as tuning bandwidth. We found that doubling the number of orientation-tuned sub-populations sampled per voxel inverted the direction of the CP data feature of the winning parameter combination for the grating dataset (Supplementary Fig. 1). This result contradicts the claim that the assumed level of granularity does not affect the qualitative pattern of results produced by Alink et al.’s model. The level of the granularity-controlling parameter *G* critically affects the nature of the signal component. It determines the extent to which the data are genuinely multivariate, rather than reflecting a single underlying dimension, such as signal strength.

We have identified an error in a recent fMRI modeling paper, from which we draw general conclusions relevant to a broad class of models. Signal strength and measurement noise influence both simulated and empirically observed correlations between brain activation patterns. These factors profoundly impact the interpretation of forward models in brain imaging. The results reported by Alink et al. hinge on assumptions neither explored nor discussed in their manuscript. Their models were not constrained by empirical estimates of key parameters determining signal and noise strength. Nor did they demonstrate robustness of their conclusions to a plausible range of noise parameters. Hence, while the instantiated forward models are useful for exploring the regimes and constraints that relate neural population responses and BOLD (blood-oxygen-level-dependent imaging) responses, they do not demonstrate that repetition suppression is best modeled by local neural scaling. Similar considerations extend more generally to the evaluation of neurobiologically minded interpretations of standard multivoxel pattern analyses^15^.

## Reporting summary

Further information on research design is available in the Nature Research Reporting Summary linked to this article.

## Supplementary information

Supplementary Information

Reporting Summary

## Data Availability

The fMRI response data relevant to this article was made available by Alink et al.^1^ and can be downloaded from the Open Science Foundation project [https://osf.io./ph26y/].

## Code availability

The code necessary to replicate Figs. 1 and 2 in our letter is based on code made available by Alink et al.^1^ from the Open Science Foundation project [https://osf.io./ph26y/]. Our modified functions can be downloaded from https://github.com/toporam/code-Ramirez-Merriam.git.
