# Supplementary material for: Forward models of repetition suppression depend critically on assumptions of noise and granularity
Source: Nat Commun. 2020 Sep 18;11:4732. doi: 10.1038/s41467-020-18315-w (PMC7501292; doi:10.1038/s41467-020-18315-w)
Supplement: Supplementary file 1 — Supplementary Information [file 41467_2020_18315_MOESM1_ESM.pdf]

Supplementary Information

Forward models of repetition suppression depend critically on assumptions of noise and granularity

Ramírez et al., Nature Communications (2020)

1. The qualitative pattern of results produced by Alink et al.'s model depends on the assumed level of granularity of the data

In p. 9 of their manuscript (section "Simulating voxel responses") Alink et al. (2018) claim that the qualitative pattern of results produced by their model is unaffected by the number of orientation-tuned neural sub-populations (or granules, or clusters) that contribute to the activity in a single fMRI voxel. This claim is incorrect, as demonstrated by previous modelling results (cf. Ramírez et al. (2014); reviewed in Ramírez (2018)) and further elucidated below. Figure S1 shows that changing the number of orientation-tuned neural granules sampled by a voxel from 10 to 18 actually *inverts* the direction of the "classification performance" (CP) data feature defined by Alink et al. In other words, even modestly increasing the number of granules per voxel can lead to a qualitatively significant change in the model's output according to Alink et al.'s criteria. This observation serves to demonstrate that granularity assumptions do affect the qualitative pattern of results produced by the class of forward models implemented by Alink et al. (2018).

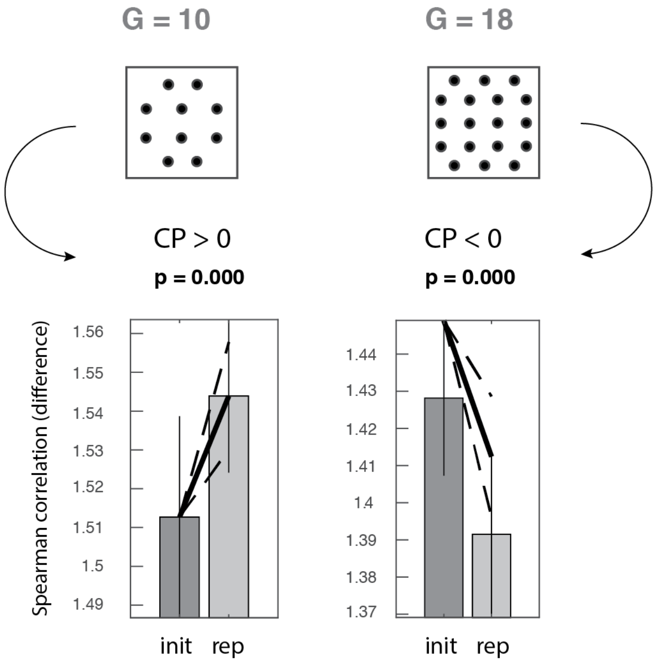

Figure S1. *Granularity assumptions affect inferences regarding neural coding based on forward models of fMRI activation patterns.* Among the six data features investigated by Alink et al., only two were found to qualitatively distinguish Experiment 1 (gratings in V1) and Experiment 2 (faces in a fusiform face-responsive region). These data-features were classification performance (CP) and amplitude modulation by selectivity (AMS). For details, see Alink et al., 2018. In this figure, simulation results are presented probing the behavior of the CP data feature only. These results will serve to demonstrate the impact of granularity assumptions on the CP data feature. The level of granularity (*G*) will be parametrized by the number of feature-tuned neural clusters assumed to reside within each voxel, following Ramírez et al. (2014). Squares on the top row represent voxels. Filled circles inside each square indicate the number of feature-tuned neural clusters (or sub-populations or *granules*) assumed to reside within each voxel in each simulation presented immediately below. The bar plot on the left shows that, when specifying a granularity level of 10, repetition suppression effects according to Alink. et al.'s model result in a relative increase of the CP data feature *after* adaptation.

This is, a relative increase of the difference between the observed within-class and between-class correlations after repeated (rep) stimulus presentations compared to initial (init) stimulus presentations. The bar plot immediately to the right, however, serves to demonstrate that changing the level of granularity from 10 to 18 *reverses* the direction of the CP data feature. This is, from a positive to a negative slope. This result plainly demonstrates that inferences regarding neural coding based on forward models of fMRI activation patterns are sensitive to assumptions about the level of granularity of the data. For a critical view on the meaning of the error bars and p-values produced by Alink et al.'s code, please refer to our letter. Init = initial, rep = repetition, CP = classification performance. WC = within class correlation, BC = between-class correlation. CP = WC – BC. See Alink et al. (2018) for further details.

1.1. *Replication of Fig S1.* The pattern of results shown on the left column of Figure S1 can be reproduced by executing Alink et al.'s function MasterScript.m (https://osf.io/ph26y/) using the default input parameters [v = 200; X = pi; cond1 = ¼*X; cond2 = ¾*X; model_type = 2; noise = 0.1; sub_num = 18] and setting Matlab's random number generator seed to the default state. In Alink et al.'s code, however, *G* is implicitly fixed to a value of eight in Line 25 of the sub-function simulate_adaptation.m. Replicating the results shown in our Figure S1 (left column) therefore require updating the granularity level of 8 chosen by Alink et al. to a value of 10. This can be done by modifying line 25 of function simulate_adaptation.m as follows:

Line 25 (Alink's model) currently reads:

```
for i = 1:length(tuning_curve_peaks);
```

Update Line 25 to Line 25 new (*G* = 10), which ought to read as follows:

```
G = 10; for i = 1:G;
```

Similarly, replicating the figure in the right column requires updating Line 25 new (*G* = 10) to Line 25 new (*G* = 18), which ought to read as follows:

```
G = 18; for i = 1:G;
```

These changes imply that, everything else being equal, instead of 8 granules (or sub-populations) per voxel, which was Alink et al.'s chosen level of granularity, either 10 (left column) or 18 granules per voxel (right column) will be simulated instead. Please note that (i) in every simulation each sub-population will be randomly assigned a preferred orientation from the same pool of eight equally-probable orientations, i.e., exactly as implemented by Alink et al., (ii) the qualitative pattern of results with *G* = 8 and *G* = 10 is identical, (iii) the results with *G* = 16 exhibit the same direction as *G* = 18, but with a less stringent p-value.

2. Model structure (and implementational details) for the reproduction of Figure 2b

Alink's published code (https://osf.io/ph26y/) basically simulates fMRI distributed response patterns associated with two stimulus gratings (45° and 135°) in early visual cortex (Expt. 2) or, alternatively, fMRI patterns associated with various stimulus faces in a fusiform face-responsive area (Expt. 1). This is done, in each case, both *before* and *after* adaptation. Such simulated fMRI brain patterns are generated for each of various candidate models of repetition suppression. By comparing the model's output with empirical measurements, these authors aimed to elucidate which candidate model of repetition suppression best explains the empirical data. See Alink et al. (2018) for a detailed description of the methods. From the perspective of the question explored by the simulations reported in Figure 2b of our letter, the only parameters in Alink et al.'s model that matter are those that define the characteristics of fMRI response patterns *before* adaptation. This is because we want to demonstrate the fact that the models being compared by Alink et al. reflect systematically different SNR regimes (see our letter for more regarding the impact of the granularity-controlling parameter on fMRI patterns). Considering that, as shown in our letter, *which* candidate models (i.e., local sharpening or local scaling) account for the observed repetition-suppression effects depends on the SNR-regime, this issue is evidently essential here. Of essence, that is, when it comes to interpreting the output of the class of forward-models implemented by Alink et al. Please note that the issues addressed here are orthogonal to additional possible questions regarding the validity of their parametrization of adaptation effects proper (see Figures S2 and S3 below for more on this matter).

Three parameters affect signal strength *before* adaptation in Alink et al.'s model: (i) tuning width (σ_{Tuning}) of the Gaussian (or circular Gaussian) functions used to model the response of each orientation-tuned neural cluster (or granule, or sub-population) to visually-presented gratings, (ii) the number of granules (*G*) sampled by each fMRI voxel, and (iii) the maximal response level of each orientation-tuned neural population. Considering that Alink et al.'s model specifies the same maximal response for all neural classes, as well as the same prevalence of each class of orientation-tuned neural clusters, this third parameter is inconsequential here and will be treated exactly as by Alink et al. As noted above, any further parameters affecting the characteristics of simulated fMRI responses *after* adaptation are irrelevant to our aims, and therefore ignored in what follows.

What distinguishes our simulation from that implemented by Alink et al.?

The first difference between our simulation and that of Alink et al. is that we have focused here on the pre-adaptation stimulus representation. A second key difference relates to the addition in our model of an additional free parameter *G* (range = [1, 512]) used to control the level of granularity of the simulated data. Parameter *G* in our model thus explicitly controls the number of orientation-tuned neural sub-populations (or clusters, or granules) assumed to reside within each simulated voxel. A third key difference is that we are specifically interested in the strength of the signal component distinguishing two simulated experimental conditions, and how it depends on σ_{Tuning} and *G*. Consequently, in the simulation reported in our Figure 2 the parameter controlling noise amplitude was set to zero—i.e., no noise was added. Please note,

however, that it is trivial to add noise, if so desired, using our script. Our implementation can add non-zero-mean centered uniformly distributed noise as well as zero-mean centered Gaussian noise (see text file included with our code for details).

What does our code do?

Running our main scripts mkFigure2fast.m and/or mkFigure2slow.m (https://github.com/toporam/code-Ramirez-Merriam.git) iterates a modified version of Alink et al.'s MainScript.m. For each iteration, a unique combination of input parameters is specified. This is, one pairwise combination of the prescribed range for σ_{Tuning} and *G*. In other words, the model will run over a range of tuning widths [σ_{Tuning} = 10 to 100, step = 10 degrees] and granularity levels [*G* = 1 to 512, step = powers of two (2^n, with n = [0, 1,…, 9])] and produce simulated fMRI response patterns associated with the two stimulus gratings (here, 45° and 90°) for each of these parameter combinations. To achieve this, Alink et al.'s functions were adapted to accept an additional parameter *G* as input and use it to control the number of orientation-tuned granules in each simulated voxel, in line with the model described by Ramírez et al. (2014). As noted above, no noise was added to the simulated fMRI patterns.

Our main function then computes the mean Euclidean distance between the fMRI-patterns associated with the two relevant gratings for each admissible pairwise combination of σ_{Tuning} and *G* within the range defined in our main script. The Euclidean distance is used here to quantify the strength of the signal component distinguishing the simulated patterns. The larger the distance, the stronger the signal component distinguishing these brain patterns. For a more detailed discussion of the complex relation between angular and Euclidean distances in the context of pattern analyses, please see Ramírez (2017), and Ramírez (2018).

2.1. *Replication of simulation results shown in Fig 2b.*

To replicate the simulation results reported in Fig. 2b of our letter, first download the necessary Matlab code [GitHub] and include all m-files in your Matlab path.

2.1.1. Fast (approximate): Run the function mkFig2LetterFast.m. This function will execute and save results of one single instantiation of the model. In other words, it will produce simulated fMRI patterns associated with both grating orientations for each combination of the free parameters σ_{Tuning} and *G*. This should take approx. 15 min in a moderately fast machine (e.g., 12-Core 2.7 GHz, 64 GB RAM) running Matlab R2016b, but could take substantially longer in a slower machine. If the flag2plot option is active, which is the default setting, the script will proceed to automatically calculate the Euclidean distance of each generated pair of fMRI pattern-vectors and plot them in the form of a surface plot. The results of this faster albeit approximate simulation are in our view sufficient to support all the essential arguments advanced in our letter. However, due to randomness integral to the structure of the model, the resulting surface will not look as smooth as that shown in our Figure2b.

2.1.2. Slower (exact): To replicate the smooth surface shown in Figure 2b, it is necessary to run the basic simulation multiple times and average the results across iterations. Before running the simulation, the Matlab random number generator is set to the 'default' state. Using different random seeds, however, should lead to hardly distinguishable results. Run the function mkFig2LetterExact.m. This function will repeat the basic simulation implemented in the "fast" version 25 times and save the results. Then, it will load these results, compute Euclidean distances for each instantiation of the model (as above), and average Euclidean distances across the 25 simulations for each combination of the parameters σ_{Tuning} and *G*. This will result in a 10 x 10 matrix of average Euclidean distances. The results are plotted using the same axis settings as the fast approach. Replicating Figure 2 takes roughly 12 hours in a moderately powerful machine.

3. Evaluation of model output and model flexibility

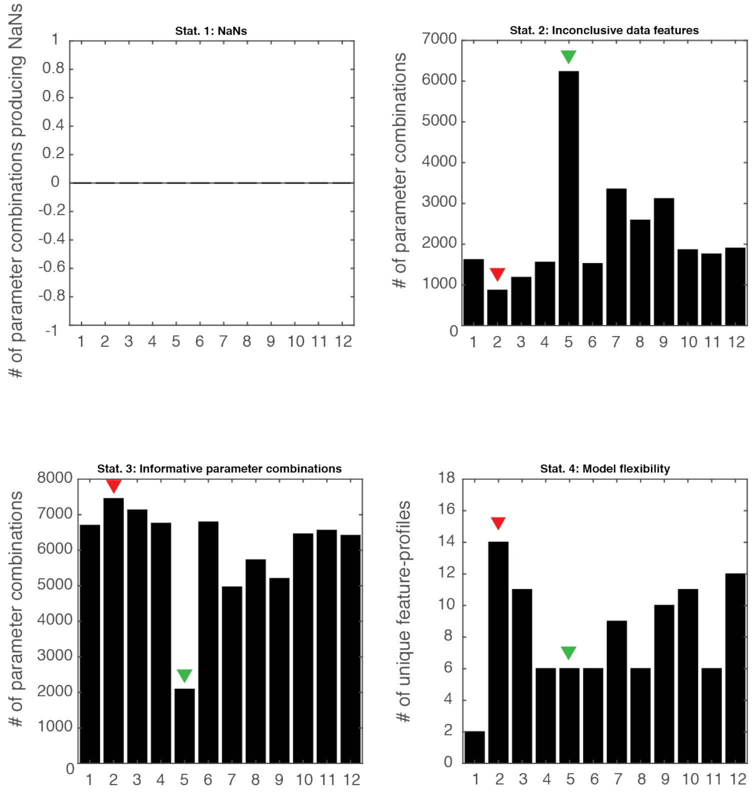

Figure S2. *Statistics describing the set of feature profiles produced by models 1-12 for Experiment 1 (faces).* On the x-axis of each plot numbers index the 12 models explored by Alink et al. Model 1 = global scaling, 2 = local scaling (highlighted with red triangles in the plots immediately above), 3 = remote scaling, 4 = global sharpening, 5 = local sharpening (shown with green triangles), and 6 = remote sharpening. Models 7-9 are the three attraction models (global, local, remote), and models 10-12 are the three repulsion models. *Statistic 1* (upper left) indicates the number of model parameter combinations producing as output a Not allowed Number (NaN). A feature profile that is a NaN is unable to fit empirical data. As noted in the upper-left plot, all models are well-specified in this case, that is, in the sense that none leads to a NaN. *Statistic 2* (top row, right panel) shows the number of model parameter combinations producing zero-valued features for each model explored. A feature profile, by definition, can't fit all the empirically observed data features if it includes a zero-valued feature—i.e., because none of the experiments led to non-significant data features. A model exhibiting higher proportions of zero-valued feature profiles is thus less likely to fit a feature profile that is known *a priori* not to include zeros. Please note that the local sharpening model exhibits by far the *highest* number of zeros, thus placing it at a disadvantage to "qualitatively fit" a random (albeit informative) feature profile. In contrast, note that the local sharpening model exhibits the *least* number of feature profiles including zeros (about 10 times less than the local scaling model). *Statistic 3* (bottom row, left) summarizes the information in the two previous panels. It indicates the total number of informative parameter combinations produced by each model—i.e., the total number of feature profiles that contain neither NaNs nor zeros. *Statistic 4* (bottom row, right) indicates the number of distinct informative feature-profiles (in the sense specified in Statistic 3) produced by each model. A model producing a larger number of distinct informative feature profiles is more likely to fit—at least in one instance—a random (albeit informative) feature profile than a model that produces a smaller set of distinctive feature profiles. Statistics 1-4 show that local sharpening is the most flexible among the models considered here. This same model also exhibits a larger number of informative—and therefore potentially "qualitatively fitting"—data features than its competitors.

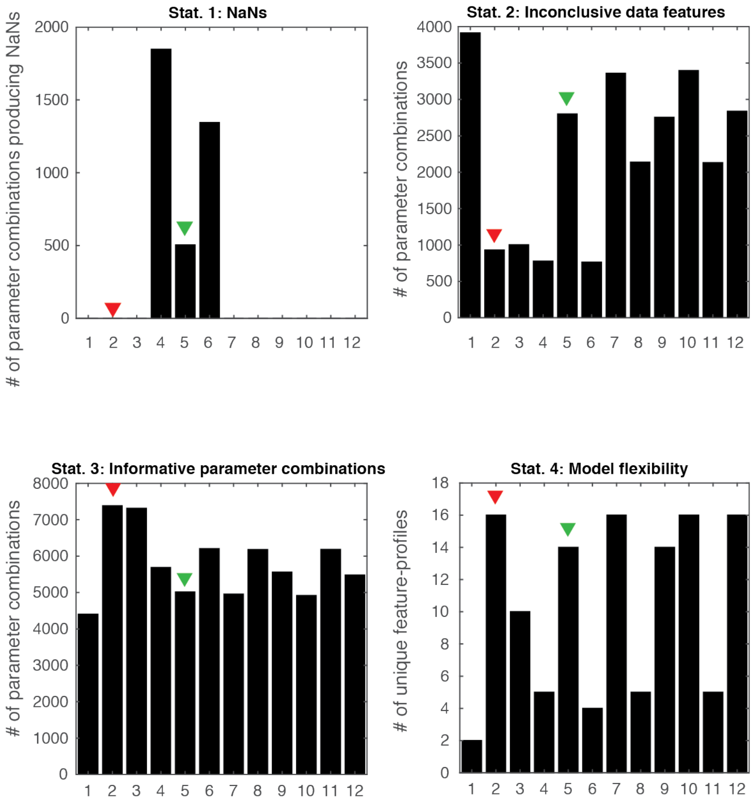

Figure S3. *Statistics describing the set of feature profiles produced by models 1-12 for Experiment 2 (gratings).* On the x-axis of each plot, numbers index the 12 models explored by Alink et al. Model 1 = global scaling, 2 = local scaling (highlighted with red triangles), 3 = remote scaling, 4 = global sharpening, 5 = local sharpening (green triangles), and 6 = remote sharpening. Models 7-9 are the three attraction models (global, local, remote), and models 10-12 the three repulsion models. *Statistic 1* (upper left) indicates the number of model parameter combinations producing as output a Not allowed Number (NaN). A feature profile that is a NaN is unable to fit empirical data. Please note that only the three variants of the local-sharpening model exhibit NaNs. This imposes an evident disadvantage for the sharpening models when compared with the remaining models. *Statistic 2* (top row, right) shows the number of model parameter combinations producing zero-valued features. A model exhibiting a higher proportion of zero-valued feature profiles is less likely to fit a feature profile known *a priori* not to include zeros. Note that the local sharpening model exhibits less zero-valued feature profiles than both the global scaling (approx. 75% less) and local sharpening models (approx. 66% less). *Statistic 3* (bottom row, left) summarizes the information in the two previous panels. It indicates the total number of informative parameter combinations produced by each model—i.e., the number of feature profiles that include neither NaNs nor zeros. *Statistic 4* (bottom row, right) indicates the number of distinct informative feature-profiles produced by each model. A model producing a larger number of distinct albeit informative feature profiles is more likely to fit a random informative feature profile than a model that produces a smaller set of distinctive feature profiles. Statistics 1-4 indicate that the manner in which the sharpening models were specified impose a penalty on these models. The local scaling model exhibits far less inconclusive data features than its main competitors. Furthermore, the local-sharpening model again proved to be the most flexible among the tested models.

4. Empirically observed Signal-to-Noise Ratios and how they compare to the Signal-to Noise-Ratio of the “winning” models reported by Alink et al.

As a first step to characterize signal strength in relation to noise strength for both the empirical data and the output of Alink et al.’s model, we adopted the definition of SNR used by Alink et al. in their response letter:

$$SNR = \frac{signal^2}{noise^2}.$$

This measure of SNR—as utilized by Alink et al.—is univariate in nature in the sense that it is blind to the spatiotemporal covariance structure associated with the signal and noise components. Model SNR estimates were thus obtained by squaring the mean response level observed across voxels for each experimental condition and dividing this value by the known variance of the Gaussian noise distribution used to generate the additive noise component. The model SNR estimates listed below correspond to those reported in Figure 1 of Alink et al.’s response letter. Please note that Alink et al., as we also did here for the sake of consistency, considered only the unadapted condition for their model SNR estimates.

Using the same definition of SNR adopted by Alink et al. for the model output, we computed comparable estimates of the SNR of the empirical fMRI data for both experiments (faces, gratings). For each subject (n = 18) and region of interest (FFR, V1) SNR was defined as the square of the mean regression weight for each experimental condition divided by the variance of the noise. The variance of the noise in each ROI was estimated by the variance of the regression weights associated with the first presentation of each stimulus class. These variance estimates were computed separately for each voxel, and then averaged across voxels. Please note that multiple initial presentations for each stimulus type were included in both datasets. For each experimental subject and ROI, one SNR estimate was thus obtained by averaging the SNR estimates for the two conditions in each experiment (face/non-face in FFR, or 45°/135° grating in V1). Below, we report the average SNR across subjects in each ROI. For completeness, we also include separate SNR estimates for each experimental condition.

Experiment 1: (event related design; faces)

eSNR

paradigm	=	face		
**empirical SNR**	**=**	**0.074**		(mean SNR across conditions 1 and 2)
meanSNRc1c2	=	0.11	0.034	(mean SNR observed for conditions 1 and 2)
medianSNRc1c2	=	0.084	0.024	(median SNR observed for conditions 1 and 2)
***lowest* fitting model SNR**	**=**	**8 – 9**		(mean SNR across conditions 1 and 2)

Comments:

1) SNR = 8 is the *lowest* SNR observed among the qualitatively fitting models reported by Alink et al. (cf. Figure 1 in Alink et al.’s reply letter). Our empirical SNR estimates for the face experiment are inconsistent with those of the models reproducing the empirically observed pattern of results.

2) The empirically observed SNR for conditions 1 and 2 varies significantly, an aspect of the data Alink et al.’s model does not accommodate.

Experiment 2: (block design; gratings)

paradigm	=	grating		
**empirical SNR**	**=**	**3.87**		(mean SNR across conditions 1 and 2)
meanSNRc1c2	=	3.90	3.83	(mean SNR observed for conditions 1 and 2)
medianSNRc1c2	=	3.52	3.64	(median SNR observed for conditions 1 and 2)
***lowest* fitting model SNR**	**=**	**53 *(96.6)***		(mean SNR across conditions 1 and 2)

Comments:

1) SNR = 53 is the *lowest* SNR observed among all the qualitatively fitting models reported by Alink et al. (cf. Figure 1 in Alink et al.'s response letter). Our empirical SNR estimates for the gratings experiment are therefore inconsistent with those of the models reproducing the empirically observed pattern of results.

2) Changing the parameter nSubj (number of subjects) in the simulation instantiated by Alink et al. from 50 (the value arbitrarily chosen by these authors) to 18 (the actual number of subjects included in experiments 1 and 2) led to a substantial increase of the minimum model SNR found to qualitatively fit the data from 53 to *SNR = 96.6*.

3) In sum, our empirical SNR estimates for the gratings experiment are inconsistent with the SNR of the models that reproduced the empirically observed pattern of results.

References

Alink, Arjen, Hunar Abdulrahman, and Richard N. Henson. 2018. "Forward Models Demonstrate That Repetition Suppression Is Best Modelled by Local Neural Scaling." *Nature Communications* 9 (1): 3854. https://doi.org/10.1038/s41467-018-05957-0.

Ramírez, Fernando M. 2017. "Representational Confusion: The Plausible Consequence of Demeaning Your Data." *BioRxiv*, September, 195271. https://doi.org/10.1101/195271.

Ramírez, Fernando M. 2018. "Orientation Encoding and Viewpoint Invariance in Face Recognition: Inferring Neural Properties from Large-Scale Signals:" *The Neuroscientist* 24 (6): 582–608. https://doi.org/10.1177/1073858418769554.

Ramírez, Fernando M., Radoslaw M. Cichy, Carsten Allefeld, and John-Dylan Haynes. 2014. "The Neural Code for Face Orientation in the Human Fusiform Face Area." *The Journal of Neuroscience* 34 (36): 12155–67. https://doi.org/10.1523/JNEUROSCI.3156-13.2014.
